# Medical Societies

**Published:** 1877-11

**Authors:** 


### Editorial.

---

### MEDICAL SOCIETIES.

One of the most interesting features of medical journals, is the department of Society Reports. In them the reader finds discussions on a great variety of subjects, many new and interesting plans of treatment proposed, and examples of their success or failure reported. These advantages are obtained, too, without the time and trouble required for reading long, prosy essays on unverified theories, whose only attraction consists in beauty of style and clever reasoning.

The meetings of societies are indispensable to the medical profession, when attended for the purposes for which they were organized. Most, if not all, of them insert in the fundamental law of their organization, such objects as, "advancement in the medical sciences," "elevation of professional standing," "increase of knowledge in scientific and practical medicine," etc. These are legitimate and praiseworthy objects, and when sought by the members in their reports and discussions, never fail to advance public and professional interests. When, however, members of a society suffer their body used by some *timid* but malignant member, for the gratification of revenge or other improper feeling, they allow it debased in the eyes of honorable men, in and out of the profession.

We will, with pleasure, publish the reports and discussions of local medical societies of this and other States, which have no journal more convenient to them—in fact, we solicit them for the benefit of our readers. As above stated, these form an interesting and useful department in medical journals; and we believe the interest felt in, and the advantages derived from such reports, would be enhanced by a more indiscriminate selection of cases by members. Some mistake the true interest of the profession, in failing to report fatal cases. Neither a desire to be considered always successful, nor the fear of criticism, as some suppose, is likely, we hope and believe, to interfere with the report of unsuccessful treatment. The record made of them is for professional men, who know well that fatal results are too often unavoidable, and that criticisms are by no means justifiable alone in these. Professional interest and humanity require the report of deaths from diseases not usually dangerous, so that if the fatal difficulty is not discovered in time, it may be anticipated by others thus warned. Give us, therefore, the safe and dangerous cases—the successful and unsuccessful treatment.

